# Untargeted metabolomics reveal rhizosphere metabolites mechanisms on continuous ramie cropping

**DOI:** 10.3389/fpls.2023.1217956

**Published:** 2023-08-22

**Authors:** Yafen Fu, Tongying Liu, Xin Wang, Yanzhou Wang, Qiulin Gong, Guang Li, Qian Lin, Siyuan Zhu

**Affiliations:** ^1^ Institute of Bast Fiber Crops, Chinese Academy of Agricultural Sciences, Changsha, China; ^2^ Selenium Resources Development and Utilization Center, Yichun Agricultural and Rural Bureau, Jiangxi, China

**Keywords:** ramie, untargeted metabolomics, rhizosphere metabolites, metabolic pathways, continuous cropping

## Abstract

Ramie is an important fiber feed dual-purpose crop in China and plays an important role in the national economy. However, ramie yield and quality can be reduced after many years of continuous cultivation. Currently, relatively little research has been conducted on rhizosphere metabolites and their pathways in continuous ramie cropping. Therefore, a healthy group (CK) and obstacle groups (XZQG, JZ, DJY, and GXD) with 8 years of continuous cultivation were selected for the study. LC-MS and GC-MS untargeted metabolomics were used to explore and analyze ramie rhizosphere metabolites and pathways. The results revealed that significant differences in the agronomic traits of ramie occurred after 8 years of continuous cultivation, with dwarfed plants and decreased yields in the obstacle groups. Metabolomic analysis identified 49 and 19 rhizosphere metabolites, including lipids, organic acids, phenols, and amino acids. In addition, four differential metabolic pathways (phenylpropanoid biosynthesis, fatty acid metabolism, amino acid metabolism, and ascorbate and aldarate metabolism) were elucidated. It was also clarified that sinapic acid, jasmonic acid, glutamine, and inositol might be the main metabolites affecting ramie continuous-cropping obstacle groups, and they were significantly correlated with ramie agronomic traits and physiological indicators. This provided important insights into the mechanisms affecting continuous ramie cropping. Accordingly, it is expected that the increase or decrease of sinapic acid, jasmonic acid, glutamine, and inositol in the soil will alleviate obstacles to continuous ramie cropping and promote the healthy development of the ramie industry in the future.

## 1 Introduction

Ramie (*Boehmeria nivea* L. Gaud) belongs to the genus Boehmeria, family Urticaceae, and is a perennial herbaceous fiber plant with persistent roots, commonly known as “Chinese grass.” China accounts for more than 90% of the world’s ramie production in terms of the area under cultivation and total production, is rich in germplasm resources, and dominates the international market. Ramie fiber is an excellent raw material for the textile industry, and its root system has a high medicinal value (Rehman et al., 2020). Under normal conditions, ramie roots can self-renew and grow for 10–20 years or even longer. However, after 3–5 years of continuous long-term cultivation, ramie renewal, root damage and root rot are inevitable. This is a major crop disorder that commonly occurs after heavy ramie cropping. Pathogenic microorganisms intensify, cell structure changes, and in severe cases, the life span shortens, and whole plant death can occur (Wang Y. et al., 2020; Zhu et al., 2018). These phenomena were typical of persistent ramie diseases. Therefore, it is important to understand the complex mechanisms of the obstacles to continuous ramie cropping.

Continuous cropping is the practice of planting the same crop or family on the same plot of land for many years. This can result in poor plant growth and development, smaller yellow leaves, increased disease, reduced crop quality, and lower yield (Lyu et al., 2022). In recent decades, experts worldwide have begun to use crop science and ecology to study the barriers to continuous cropping at greater depths. For example, continuous cropping of soybean leads to the frequent occurrence of soil-borne fungal diseases (Song et al., 2022); long-term continuous cropping of strawberry leads to unfavorable inter-root soil conditions and reduces soil physicochemical properties and enzyme activities (Li et al., 2018); and continuous cropping of oil flax reduces the diversity of soil bacteria and affects the structure of the bacterial community (Wang L. G. et al., 2022). Wang Y. et al. (2020) found that continuous ramie cropping negatively affected ramie itself, affecting the physiological, biochemical, and root microbial community diversity. According to the results of Bai (2017), potential chemosensitive substances in ramie have a negative impact on ramie itself, affecting its physiology, biochemistry, and root microbial community diversity.

The effects of continuous cropping are significant, while two categories of factors are reported in the literature regarding the formation of disorders in crops: abiotic and biotic. Abiotic factors are mainly considered as altered physical and chemical properties of the soil, improper application of fertilizers and herbicides, soil nutrient imbalance, poor soil structure, and irrigation practices (Subhashini and Kumar, 2019; Wang S. et al., 2020). Biotic factors include mainly many biotic and abiotic factors, such as changes in microbial communities (Dong et al., 2017), enrichment of soil-borne plant pathogens (Liu et al., 2014), decreased soil enzyme activity (Fu et al., 2017), changes in soil physicochemical properties (Kaur and Singh, 2014; Perez-Brandan et al., 2014; Li et al., 2018), and plant self-toxicity (Van Wyk et al., 2017). In recent years, research on cropping barriers has focused on the chemosensitization of plant autotoxic substances, which is defined as a biological phenomenon in which an organism affects the germination, growth, survival, and reproduction of other organisms in the same community by producing one or more biochemical substances (Xu et al., 2012; Huang et al., 2021). The long-term continuous cropping of soybean leads to the accumulation of harmful secondary metabolites (phenolic acids, benzene, and esters) in root secretions, resulting in soybean autotoxicity and reduced yield and quality (Cui et al., 2018). Previous studies in our laboratory (Wang Y. et al., 2020) found that metabolites such as cysteine, glycine, uracil, and malonic acid were disturbed after continuous ramie cropping and were used as risk markers. The present study builds on these findings by selecting a healthy control group and four species that developed continuous crop disorder to further explore the changes in ramie rhizosphere metabolites and their mechanisms in various continuous cropping groups.

Metabolomics is an important component of systems biology that identifies the full spectrum of detectable metabolites present in biological systems (Wang R. et al., 2022). It is a powerful bioanalytical tool widely used for qualitative and quantitative studies of metabolites in various test samples. Although untargeted gas chromatography-mass spectrometry (GC-MS) and liquid chromatography-mass spectrometry (LC-MS) have been widely used to detect many plant metabolites (Dhawi et al., 2016; Son et al., 2016), few studies have reported the application of metabolomics to explore crop-linked disorders in ramie. Therefore, in this study, we used an untargeted metabolomics approach that combines LC-MS and GC-MS. We selected a healthy group after 8 years of cultivation (CK) and ramie varieties (XZQG, JZ, DJY, and GXD) that developed continuous crop disorder as experimental materials to investigate the effects of continuous and non-continuous group treatments on ramie biomass, rhizosphere soil physicochemical properties and rhizosphere Metabolites and metabolic pathways. These results provide a comprehensive understanding of the stress state of ramie after continuous cropping. It may provide important insights into the mechanisms of the obstacles to continuous ramie cropping and may help to alleviate the effects of continuous ramie cropping by regulating related metabolites and metabolic pathways to promote the healthy development of the ramie industry.

## 2 Materials and methods

### 2.1 Plant materials collection

Ramie cultivars were planted in a test field at the Institute of Bast Fiber Crops, Chinese Academy of Agricultural Sciences, Changsha, China. Five ramie germplasm resources, comprising a healthy (CK) and four damaged varieties (XZQG, JZ, DJY, and GXD), that had been cultivated for 8 years and provided the names, collection sites, categories, and resequencing data summary in **Table S1**. The experiment began in March 2015 and lasted until June 2022, during which the plot was cultivated using a continuous cropping system (3 seasons per year for 5 consecutive years). All the plots were cultivated under the same cropping system and conditions, including annual sowing time, harvest time, fertilization, and irrigation methods. The rhizosphere soil samples were collected on August 3, 2022, from successive crops and then mixed thoroughly. The mixed soil samples from each group were divided into two parts for GC-MS and LC-MS analyses (6 samples were randomly selected for both, n = 6), and 30 ramie rhizosphere soil samples were prepared for each of these two analyses. All collected samples were stored at −80°C immediately after preparation.

### 2.2 Plant agronomic traits and physiological measurements

The measured agronomic traits included stem length (SL, cm), stem diameter (SD, mm), bark thickness (BT, mm), bark weight (BW, kg), Fiber yield (FY, g) and fiber yield (FOR, %). Following simultaneous sampling, enzyme activity was measured in the collected rhizosphere soil samples. The activities of the soil enzymes acid phosphatase (S-ACP), neutral phosphatase (S-NP), urease (S-UE), and sucrase (S-SC) were measured using ELISA kits (MLBIO, Shanghai, China) as describe by Zhao et al. (2022). Soil pH was measured using a pH after the samples were dried at 105°C for 24 hours to determine soil moisture content. Soil pH was determined using a calibrated pH meter (soil: solution ratio of 1:2.5).

### 2.3 Sample preparation

For untargeted metabolite analysis, each sample of approximately 0.5 g was sonicated for 2 min by adding 1 mL of pre-cooled solvent (methanol/water 1:1 (v/v), containing L-2-chlorophenylalanine, 2 μg/mL), followed by centrifugation (10 min at 4°C and 12000 rpm) and 1 mL was transferred to a sample vial. Next, 200 μL of methanol-water (1:4 (v:v)) was redissolved for 3 min, and the reaction was carried out at −40°C for 30 min. The sample was then centrifuged (10 min at 4°C and 12000 rpm), and 150 μL of the supernatant was aspirated, filtered through a 0.22 μm organic phase pinhole filter, and transferred to a vial for LC-MS analysis. In parallel, a 100 μL aliquot of the supernatant was transferred to a glass sampling vial, and then 80 μL of methoxypyridine hydrochloride solution (15 mg/mL) was added, vortexed for 2 min, and then incubated at 37°C for 60 min. Subsequently, 50 μL of BSTFA derivatization reagent and 20 μL of hexane were added, vortexed for 2 min, and then incubated at 70°C for 60 min. Samples were incubated at 25°C for 30 min and subjected to GC-MS metabolomic analysis.

### 2.4 LC-MS-based untargeted metabolomics analysis

LC-MS setup: The injection volume was 10 μL. The separation was performed on an HSS T3 C18 column (100 mm × 2.1 mm × 1.8 μm, Waters) with the column temperature maintained at 50 °C. The gradient elution program was set as follows: 0-2 min, 100% A; 2-11 min, 0%-100% B; 11-13 min, 100% B; 13-15 min, 0%-100% A. The Q-TOF mass spectrometer was operated in positive and negative ion modes. ESI source temperature: 120°C; desorption temperature: 450°C; desorption gas: 800 L/h; cone gas: 50 L/h; TOF mass range: 50-1200 Da; scan time: 0.2s. The LC-MS metabolic profiles were acquired using an ACQUITY UHPLC system (Waters Corporation Milford, USA) with an AB SCIEX Triple TOF 5600 system (AB SCIEX, Framingham, MA, USA) in the ESI positive and negative ion modes. The QC samples were injected at regular intervals (every 6 samples) throughout the analysis to provide a data set to assess reproducibility. The raw LC-MS data were provided by Luminous (Shanghai, China), and metabolites were identified primarily based on RT m/z pairs and tandem mass spectrometry (MS/MS) spectra, HMDB (https://hmdb.ca/), LIPID MAPS (https://lipidmaps.org/), and a self-constructed database. The data matrix, including 3D datasets of m/z, RT peaks, and intensities, was exported as an Excel file for further analysis.

### 2.5 GC-MS-based untargeted metabolomics analysis

GC-MS setup: GC-MS analysis was performed using a hybrid quadrupole-Orbitrap GC-MS/MS system (Q Exactive GC, ThermoFisher). The sample volume injected was 1 μL at a carrier gas flow rate of 1 mL/min of helium. The oven temperature was initially set to 50°C and held for 1 min, ramped up to 100°C at 10°C/min, and immediately ramped up to 200°C at 10°C/min and held for 1 min, then ramped up to 320°C at 10°C/min. The ion source temperature was set to 230°C. The scan range was 50.0 to 650.0 m/z, and the mass spectrum was acquired in electron ionization mode (70 eV).

The raw data from the GC-MS analysis were converted to ABF (Analysis Base File) format using analysis base file converter software, and peak detection, deconvolution, alignment, and filtering were performed to identify the metabolites based on the LUG database ((Untargeted database of GC–MS from Lumingbio)), removing all internal standards and false positive peaks. The raw data matrix of the sample information, retention times, and peak intensities was then exported to an Excel file for further analysis.

### 2.6 Multivariate and univariate analysis and biological pathway analysis

Partial least squares discriminant analysis (PLS-DA) is a supervised clustering method that uses multiple linear regression techniques to maximize separation between groups and to help understand which variables carry class separation information (Yang et al., 2018). PLS-DA was performed on the LC-MS and GC-MS results using online resources (http://www.metaboanalyst.ca/). The variable importance (VIP) in the projection is the weighted sum of squares from the PLS-DA analysis, which indicates the importance of a variable to the overall model. Metabolites with a VIP > 1 and *P*< 0.05were considered significant. Univariate (one-way ANOVA) analysis of LC-MS/MS results was performed using an online resource (http://www.metaboanalyst.ca/) (Miska et al., 2021). Biological pathway analysis was performed on all metabolites data using MetaboAnalyst 5.0. The calculated impact value threshold for pathway identification was set to 0.1 (Li et al., 2022).

## 3 Results

### 3.1 Effects of continuous soil cropping on ramie

**Figure 1:**
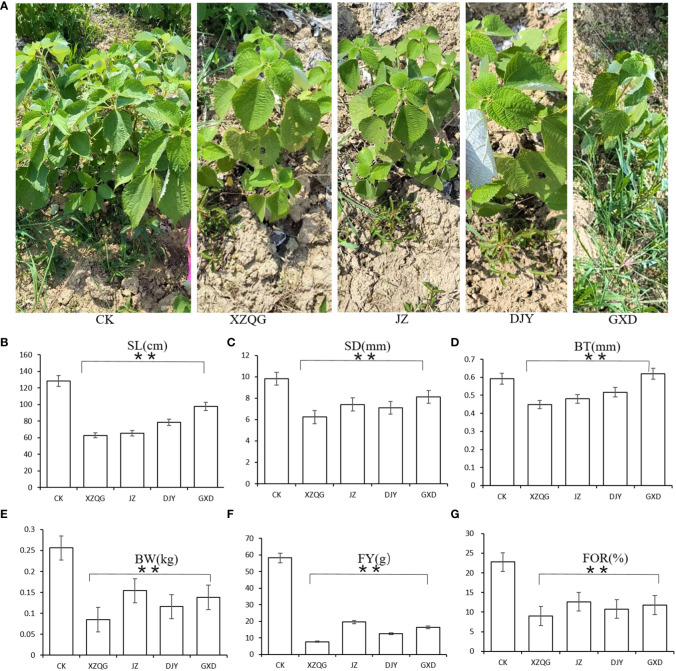
**(A)** Diagram of ramie after five years of continuous cropping. From left to right: CK; XZQG; JZ; DJY; GXD. **(B)** SL. **(C)** SD. **(D)** BT. **(E)** BW. **(F)** FY. **(G)** FOR. **indicates a significant difference at the *p* <0.001 level.

After 8 years of continuous cropping of ramie plants, the ramie grown in the 1-year rhizosphere soil was very robust (defined as the CK group), whereas the ramie grown in the four varieties in the continuous cropping had weak growth and low yield (defined as the obstacle groups). The values of plant height, stem diameter, bark thickness, weight, and weight of fresh and dry fibers were lower in the impaired group than in the healthy group (*P*<0.001; **Figure 1**). These findings confirm that there is a great difference between growing the CK and obstacle groups in the same field.

Most of the pH values tested in the 8-year rhizosphere soil were bellow pH 7, indicating an acidic soil. At the same time, there were significant differences in the rhizosphere soil enzyme activities among the different planting years. The activities of polyphenol oxidase, neutral phosphatase, and urease were lower than the CK group, whereas the urease activity increased (**Table 1**). Meanwhile, it was found that there were significant differences in soil enzyme activities among different cropping years (P < 0.001, **Figure 1**). The activities of soil acid phosphatase (S-ACP) and soil neutral phosphatase(S-NP) decreased with increasing years of continuous cropping and were all lower than those of the CK group, whereas soil urease (S-UE) activity increased after 8 years of continuous cropping (**Table 1**).

**Table 1:** Differences in soil chemical parameters between the CK and Obstacle group.

	CK	XZQG	JZ	DJY	GXD	*P*-value
**PH**	7.03 ± 0.10	5.42 ± 0.02	5.24 ± 0.02	4.79 ± 0.03	5.82 ± 0.02	<0.001
**S-ACP (nmol/h/g)**	1155.40 ± 7.64	2096.21 ± 4.58	2482.29 ± 3.51	3067.51 ± 6.54	1697.94 ± 2.64	<0.001
**S-NP (nmol/h/g)**	842.09 ± .00	158.99 ± 2.65	547.69 ± 2.00	283.19 ± 2.52	628.19 ± 1.00	<0.001
**S-UE (μg/d/g)**	52.72 ± 1.15	67.76 ± 1.00	70.16 ± 4.16	57.68 ± 1.00	87.68 ± 1.53	<0.001
**S-SC (mg/d/g)**	8.00 ± 0.15	6.14 ± 0.15	5.93 ± 0.10	6.54 ± 0.01	5.97 ± 0.06	<0.001

Values followed by different letters were significantly different according to Duncan’s multiple range tests (P <0.001).

### 3.2 Metabolic profiles analyzed by GC–MS and LC–MS

**Figure 2:**
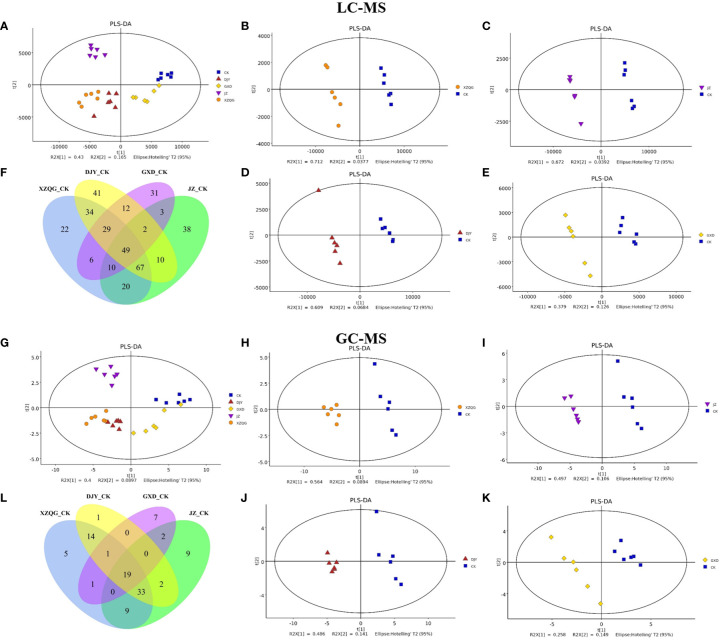
Metabolomic analysis of ramie continuous crop obstacle groups. **(A)** PLS-DA score plots of LC-MS between CK group and all obstacle groups **(B-E)** PLS-DA score plots of LC-MS between CK group and each obstacle group, **(F)** LC-MS metabolite Venn diagram; **(G)** PLS-DA score plots of GC-MS between CK group and all obstacle groups **(H-K)** PLS-DA score plots of GC-MS between CK group and each obstacle group, **(L)** GC-MS metabolite Venn diagram.

Metabolite profiling was performed on the rhizosphere soil samples of the CK and four obstacle groups (XZQG, JZ, DJY, and GXD) using LC-MS and GC-MS. In the LC-MS analysis, PLS-DA was used to identify the metabolites that led to the separation between the CK and obstacle groups. The results showed that the different groups underwent relatively clear clustering, and there was no significant sample overlap, suggesting that the metabolites detected using LC-MS differed significantly between species (**Figure 2A**). Furthermore, when the CK group was compared with each obstacle group, the CK group and the other groups were located within a 95% Hotelling’s T^2^ ellipse in the PLS-DA models, showing a clear separation (**Figures 2B-E**). A further 7-fold internal cross-validation and 200 permutation tests were performed to assess these models’ predictive accuracy and statistical significance. The slopes of the R2Y and Q2Y straight lines were found to be very close to the horizontal straight line for each group compared to the CK group (**Figure S1**). In the GC-MS analysis, the CK group in the PLS-DA models showed the same significant separation from the other total obstacle groups (**Figure 2G**), as well as when compared individually (**Figures 2H-K**). The blue regression line at the Q2 point was below zero with the vertical axis in the 200 permutations 1 component test, and these PLS-DA models showed a low overfitting risk. These results suggest that the PLS-DA models can identify enriched metabolites that differ between the CK and obstacle groups (**Figure S2**).

### 3.3 Changed metabolites in ramie soil between the CK and obstacle groups

**Figure 3:**
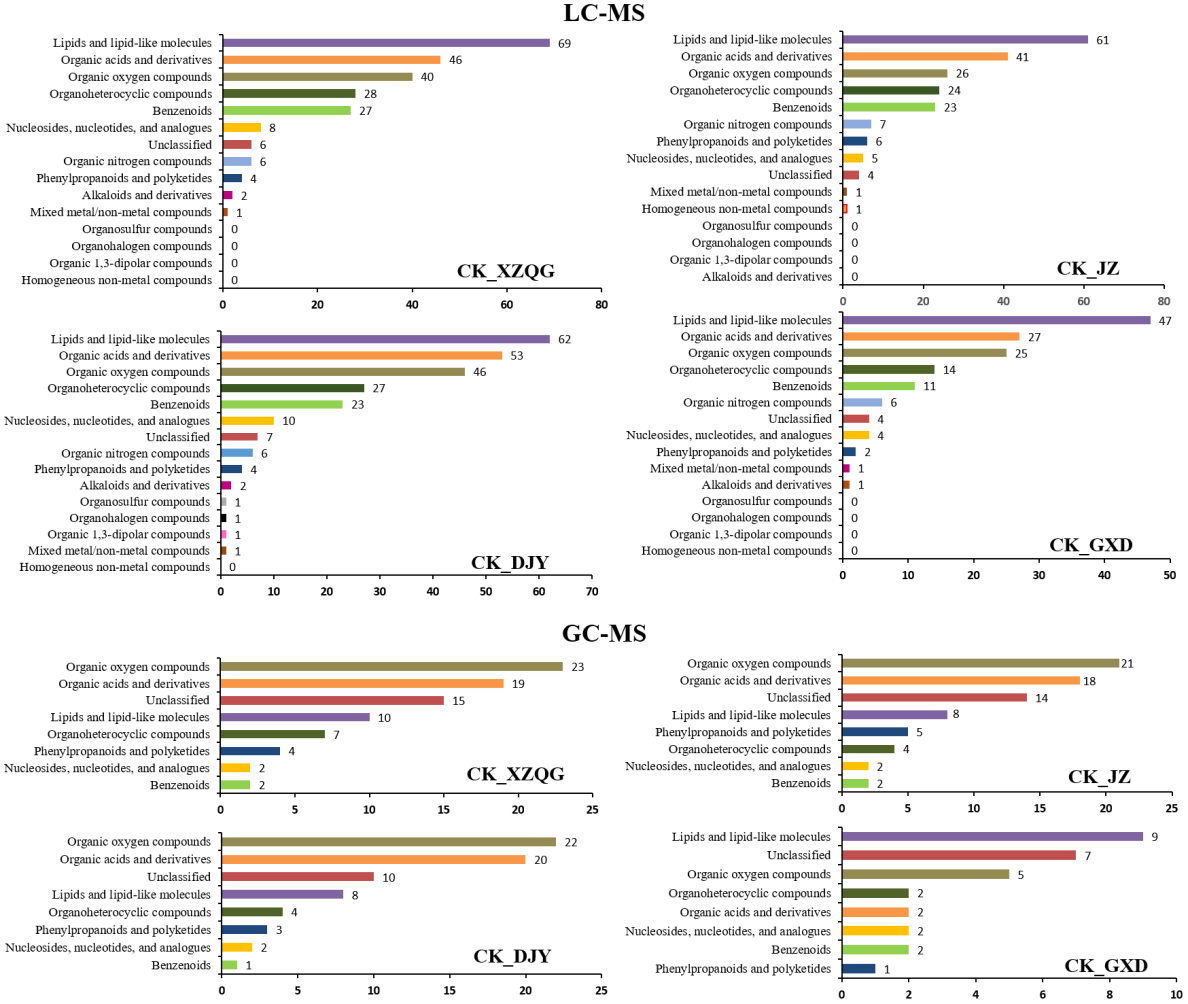
Analysis of differentially expressed metabolites classification statistics using LC-MS and GC-MS.

A comprehensive LC-MS and GC-MS untargeted metabolomics approach was used to analyze the overall metabolic profiles of continuous ramie crop soil samples. We selected metabolite peaks with a coefficient of variation (CV) <15% in 30 quality control samples and selected metabolites with large differences based on the VIP value of the PLS-DA model (VIP>1) and the corrected *p*-value of the Student’s t-test (*p*-value < 0.05). As shown in **Figure 2**, the differentially expressed metabolites produced under different explosive exposures in the LC-MS analysis were mainly lipids and lipid-like molecules (47–69), organic acids and their derivatives (27–53), and organic oxygen compounds (25–46), whose abnormal differences in metabolites may be responsible for cytotoxicity. Simultaneously, when the CK group and the obstacle groups were analyzed using LC-MS untargeted metabolomics, 49 metabolites were identified and quantified (**Figure 2F**), of which 27 were upregulated, and 22 were downregulated (VIP>1; **Table S2**). In GC-MS analysis, the differential metabolites mainly included organic oxygen compounds (5–23), organic acids and their derivatives (2–20), lipids and lipid-like molecules (8–10), organic heterocyclic compounds (2–7), phenylpropanoids and their derivatives (1–5), among others (**Figure 3**), while the CK group combined with obstacle group analysis revealed that 19 metabolites were quantified (**Figure 2L**); 12 metabolites showed an increasing trend and 7 metabolites showed a decreasing trend (VIP>1, **Table S3**).

### 3.4 Metabolic pathways of differential abundant metabolites

**Figure 4:**
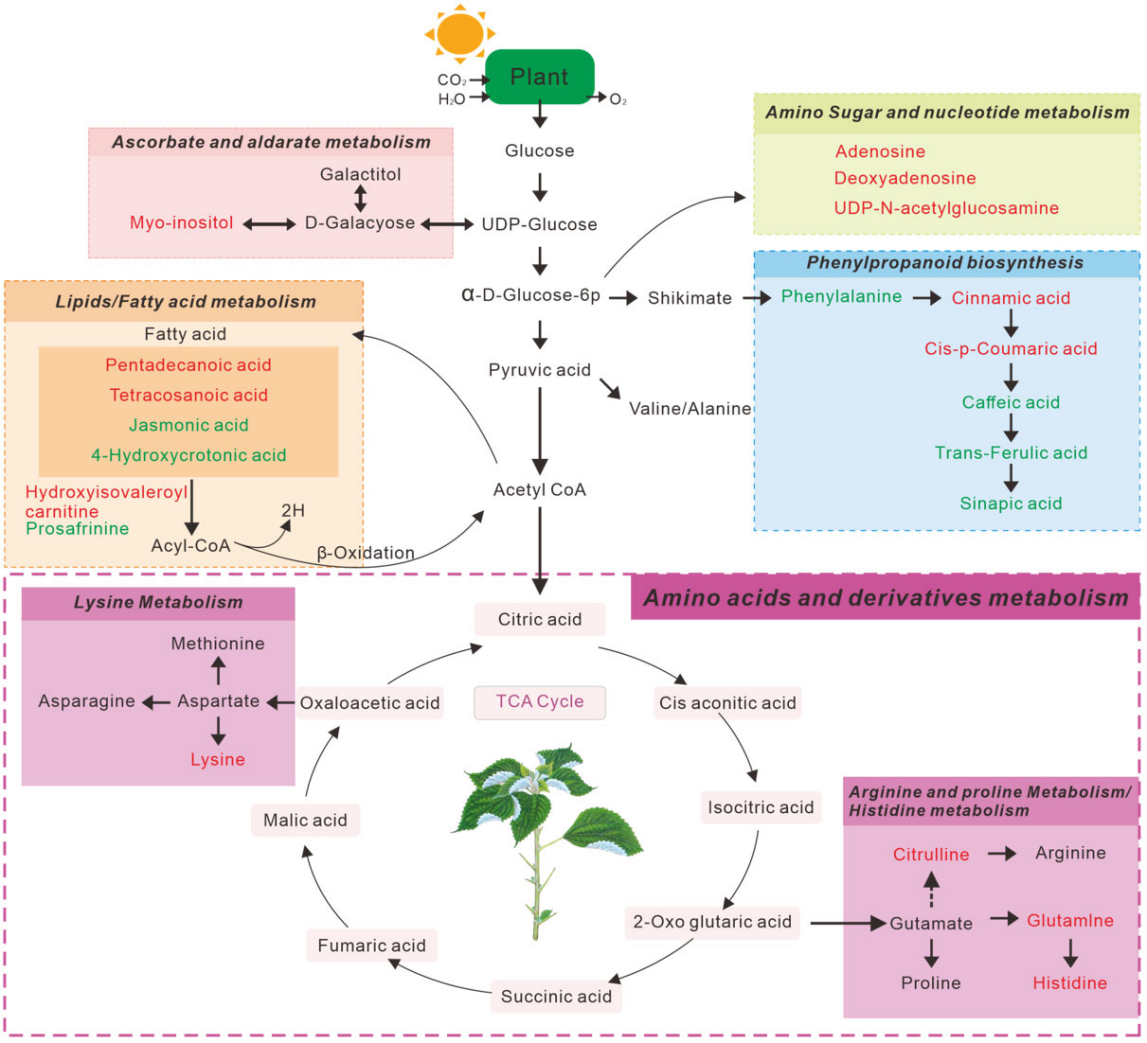
Schematic diagram of the main metabolic pathways affected by ramie rhizosphere soil metabolites. The red text represents metabolites with increased concentrations, and green text represents metabolites with decreased concentrations.

The above significantly altered metabolites are involved in several important metabolic pathways. Subsequently, we searched 49 metabolites from the LC-MS (**Figure 2F**) and 19 metabolites from the GC -MS (**Figure 2L**), determined using the Venn diagrams in the KEGG database, which revealed 10 metabolic pathways **Tables S2, S3**) that were altered in continuous ramie soil crops. Simultaneously, we performed enrichment analysis using MetaboAnalyst 5.0 to characterize the affected biological pathways and constructed as chematic diagram showing that there were 20 differential metabolites of interest in these differentially enriched metabolic pathways (**Figure 4**). These differentially enriched metabolic pathways were classiedas phenolic, lipid, aminoacid, nucleoside, ascorbate, and aldarate metabolic pathways.

**Figure 5:**
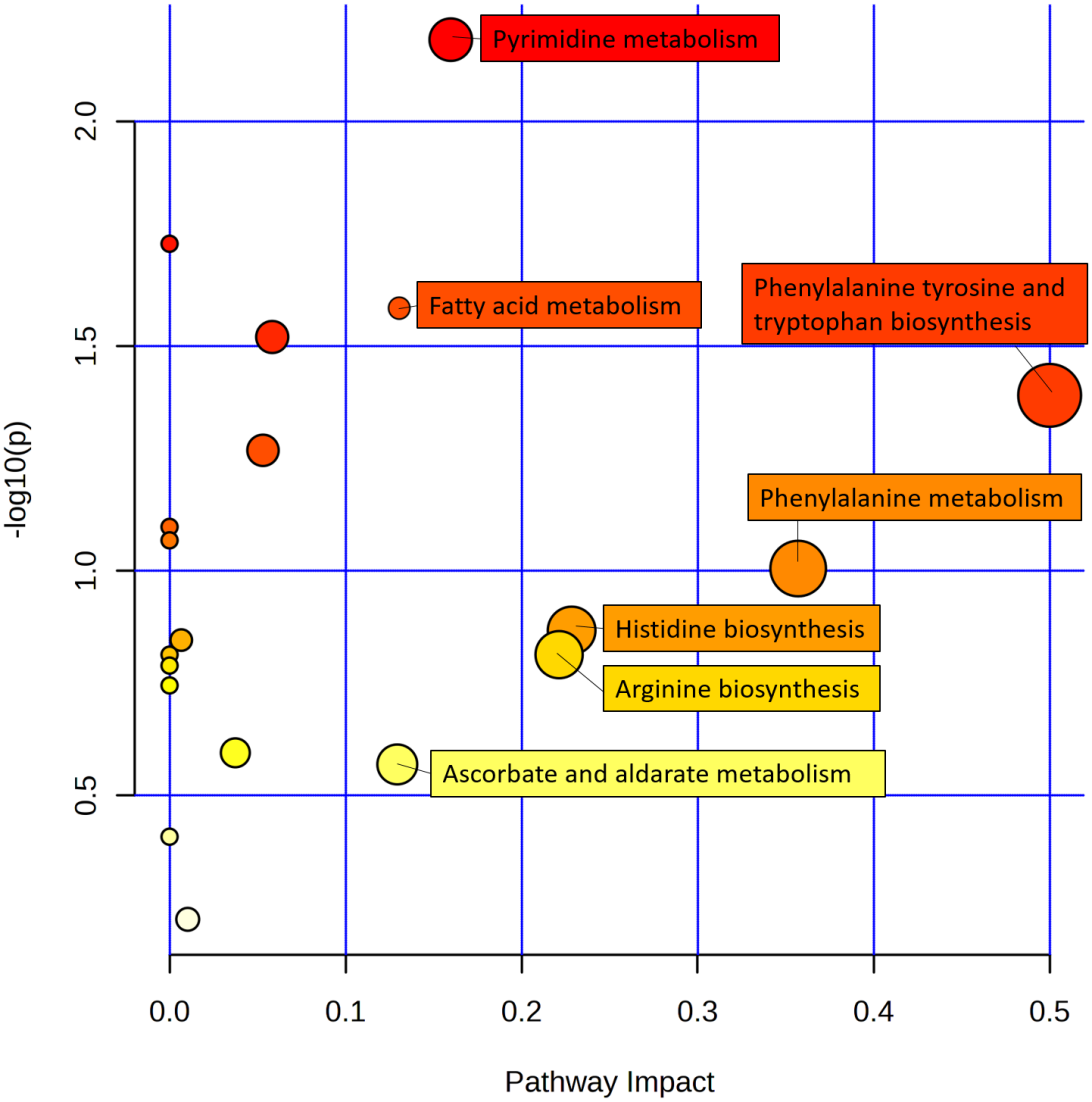
Schematic metabolome profiles of the significant metabolic pathways in ramie after continuous cropping. Each bubble in the figure is a metabolic pathway, with the x-axis indicating pathway enrichment and the y-axis indicating pathway impact. The size and color represent the enrichment and impact values of the major pathway, respectively.

Among these differentially enriched metabolic pathways, seven had impact values >0.1, which is the correlation threshold following pathway enrichment and topological analysis. Based on negative log (*P*) and impact values, we identified ramie as a significantly correlated pathway in soil secretion for phenylalanine, tyrosine and tryptophan biosynthesis; phenylalanine metabolism; pyrimidine metabolism; fatty acid metabolism; histidine biosynthesis; arginine biosynthesis; and ascorbate and aldarate metabolism. Their impact values were 0.5, 0.36, 0.23, 0.23, 0.16, 0.14, 0.13, respectively (**Figure 5**).

### 3.5 Associations between metabolic changes and physiological parameters

**Figure 6:**
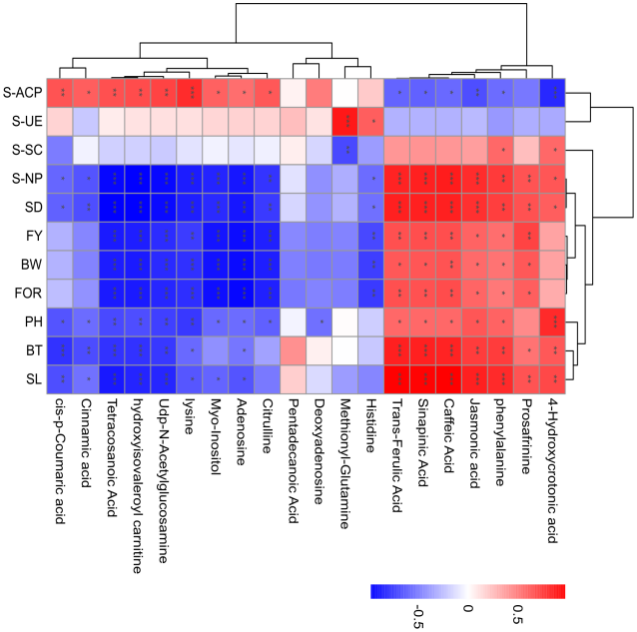
Association network analysis between metabolite changes and physiological parameters in continuous ramie cropping soil. The absolute values of Pearson correlation coefficients above the threshold are shown (*P*<0.01), and the correlation levels are indicated as red (positive correlation) or blue (negative correlation), with darker colors indicating stronger correlations. Symbols provided on bars: *: *P* < 0.05; **: *P* < 0.01; ***: *P* < 0.001.

A Spearman correlation analysis was performed to evaluate the relationship between the 20 annotated metabolites and 11 physiological parameters in continuous ramie cropping soils. Only the correlations with P < 0.05 were highlighted in the heat map (**Figure 6**). In phenylpropanoid biosynthesis following continuous ramie cropping, caffeic acid, sinapinic acid, trans-ferulic acid, and phenylalanine were positively correlated with SL, BT, SD, BW, FOR, FY, PH, S-NP, and S-SC, and negatively correlated with S-ACP and S-UE, suggesting that phenolic acids could be used in ramie metabolic pathways. Regarding fatty acid metabolism, tetracosanoic acid, jasmonic acid, and 4-hydroxycrotonic acid were positively correlated with agronomic traits (SL, BT, SD, BW, FY, and FOR) and negatively correlated with S-UE, indicating that highly expressed lipid metabolites may disrupt normal plant growth homeostasis and adversely affect plants. In amino acid and derivative metabolism, L-histidine, L-lysine, and citrulline were positively correlated with S-UE and S-ACP and negatively correlated with other physiological indicators, which demonstrated that soil urease activity and soil acid phosphatase activity were enhanced after years of continuous ramie cultivation. Plant antioxidant capacity was also enhanced. Myo-inositol, which is derived from ascorbate and aldarate metabolism, has important antioxidant effects and can scavenge reactive oxygen species and free radicals. Accordingly, myo-Inositol was positively correlated with S-ACP, S-SC, and S-UE and negatively correlated with other physiological indicators, implying that this metabolite may also be an effective metabolite for improving the environment during continuous cropping.

## 4 Discussion

The current data suggest that ramie may have undergone strong metabolic changes following 8 years of continuous cropping. Identifying these metabolites could be useful in developing potential chemical markers for monitoring or even predicting whether ramie plants are affected by continuous cropping. In the present study, a combination of metabolomic analyses focusing on 49 metabolites in LC-MS and 19 differential metabolites in GC-MS and their significantly related metabolic pathways, revealed significant differences with ramie agronomic properties, enzyme activity, and other indicators, which provided new insights into the barrier response of soil metabolites after continuous ramie cropping.

### 4.1 Phenylpropanoid pathway

The phenylpropanoid pathway, a classical metabolic pathway associated with the induction of resistance, can inhibit or scavenge ROS in cells under biotic or abiotic stress, thereby protecting proteins, membrane lipids, DNA, enzymes, and other organellar components from severe damage (Chen et al., 2006; Xu et al., 2019; Oliva et al., 2021; Wang et al., 2021). Notably, in the present study, some compounds associated with phenylpropanoid biosynthesis, such as cinnamic acid and cis-p-coumaric acid, increased the relative abundance of ramie growth in the obstacle group by 1–3-fold compared to the CK group (*P*<0.05), which may be due to an overreaction of stress to ROS, producing secondary metabolites that protect themselves from oxidative damage by reactive oxygen species generated by adversity stress. This is similar to the findings of Zhao et al. (2018), who found that phenylalanine and 4-hydroxycinnamic acid levels were enhanced in maize under adverse stress. Interestingly, phenylalanine, as well as derivatives of hydroxycinnamic acid (caffeic acid, trans-ferulic acid, and mustardic acid), showed a significant downward trend relative to the CK group, especially the downstream phenylpropanoid pathway compound (mustardic acid), which showed a 5–10-fold decrease in relative abundance (**Table S4**). It is well known that aromatic acids are important metabolites for the study of phenylpropanoid metabolism involved in the regulation of plant growth and development and response to adversity. They are widely present in the plant kingdom, where they possess antioxidant, antibacterial, anti-inflammatory, anticancer, and anxiolytic activities (Nićiforović and Abramovič, 2014). This suggested that the metabolism of the phenylpropanoid biosynthetic pathway was disrupted after ramie crop succession. Meanwhile, correlation analysis of physiological indicators of ramie showed that phenylalanine, caffeic acid, sinapinic acid, and trans-ferulic acid in the phenol propane pathway were positively correlated with SL, BT, SD, and S-NP and significantly correlated with SL with correlation coefficients of 0.814, 0.982, 0.961, and 0.972, respectively (**Table S4**). In addition, the metabolomic analysis results showed that these metabolites were downregulated, indicating that these phenolic acid products in ramie inter-root metabolites seriously affected the normal growth of ramie plants and led to poor plant growth. This indicates that these metabolites can promote plant growth at certain concentrations. Interestingly, cinnamic acid and cis-p-coumaric acid in the phenol-propane pathway were negatively correlated with SL, BT, PH, SD, and S-NP; in particular, cinnamic acid and cis-p-coumaric acid were correlated with BT with the highest correlation coefficients of -0.683 and -0.781, respectively (**Table S4**). These two metabolites were upregulated in the metabolomic analysis, indicating that high concentrations of phenolic acids can inhibit ramie growth and lead to the development of succession disorders. This result was mirrored in a similar study by Li et al. (2017), in which cinnamic acid had an inhibitory regulatory effect on cucumber morphogenesis and development. In conclusion, while some metabolites (phenylalanine, caffeic acid, sinapinic acid, trans-ferulic acid) were depleted to protect the plants from adverse stress, the intermediates of phenylalanine metabolism (cinnamic acid, cis-p-coumaric acid) appeared to accumulate, indicating that the accumulation and depletion of secondary metabolites in ramie are both manifestations of ramie’s response to continuous ramie cropping and subsequently ensure the normal growth of ramie.

### 4.2 Fatty acid metabolism

Fatty acids, which are components of cell membranes, are essential for maintaining cell fluidity and integrity under various stress levels (Raval et al., 2017). Interestingly, the fatty acids that were upregulated in the CK group compared with those in the obstacle groups were mainly saturated fatty acids, including pentadecanoic acid and tetracosanoic acid, some of which are metabolites derived from the malonyl-CoA precursor of flavonoid biosynthesis (Lyu et al., 2018). Raval et al. (2017) also found a higher accumulation of palmitic and stearic acids in an adversity study. This could indicate that adversity induces changes in fatty acid composition. However, unsaturated fatty acids make membranes more susceptible to ROS attack (Paupière et al., 2014). In the current study, unsaturated fatty acids (jasmonic acid and 4-hydroxycrotonic acid) were significantly down-regulated by 1–3 fold relative to the CK group, demonstrating that the saturated level of fatty acids seems to have a critical effect on membrane stability and normal growth of ramie plants. Simultaneously, jasmonic acid and 4-hydroxycrotonic acid were positively correlated with agronomic traits, such as SL, BT, PH, SD, and S-NP, especially in the jasmonic acid variable with SL, BT, SD, and S-NP, with significant correlations of 0.857, 0.749, 0.739, and 0.811, respectively (**Table S4**). These results are similar to the view that jasmonic acid initiates the plant immune defense response and activates related genes and metabolites (Zhang et al., 2017). Notably, jasmonic acid is an endogenous growth regulator present in higher plants with physiological effects, such as inhibition of plant growth, promotion of leaf senescence, and enhancement of resistance. More strikingly, jasmonic acid-like JA-mediated signaling pathways are closely related to plant resistance and can induce the expression of resistance genes, effectively mediating plant defense responses to pathogenic bacteria, herbivores, and abiotic stresses, inducing the expression of a series of defense genes, synthesis of defense response chemicals, and modulation of plant immune and stress responses (Han, 2017). Preliminarily, jasmonic acid may be one of the factors affecting ramie succession.

### 4.3 Amino acid and derivatives metabolism

The accumulation of amino acids and other nitrogenous compounds has been observed in many studies on plants exposed to adversity (Sanchez et al., 2008). The current study showed that the altered amino acids were mainly involved in the metabolism of arginine, proline, histidine, and lysine. Specifically, lysine, citrulline, histidine, and methionyl glutamine were upregulated, especially methionyl glutamine was upregulated by a factor of 10–40 (**Figure S3**). Glutamine is an important form of nitrogen present in plants and is a precursor for the intracellular biosynthesis of many important substances, such as nucleic acids, nucleotides, amino sugars, and proteins, as well as a carrier of nitrogen during ammonia and urea synthesis *in vivo*. Glutamine is also an important component of the nitrogen cycle; in rapidly multiplying and differentiating cells and tissues, it is preferentially selected as the main energy source and plays a key role in nitrogen assimilation, recycling, transport, and storage. (Zhao et al., 2018). The increase in glutamine and glutamate levels was also consistent with previous reports showing that glutamine is significantly upregulated and elevated in response to adverse stress (Rizhsky et al., 2004). In addition, the correlation between glutamine and S-UE was significant, with a correlation coefficient of 0.889 (**Table S4**), indicating that glutamine affected urease activity in ramie soil and was closely related to soil nutrient regulation. In conclusion, the increase in glutamine may imply that ramie was under adverse stress after continuous cropping and needed to continuously enhance nitrogen assimilation, cycling, transport, and storage.

### 4.4 Ascorbate and aldarate metabolism

The ascorbic acid pathway is critical for regulating plant growth, inducing flowering, delaying senescence, scavenging self-reactive oxygen species and free radicals, reducing damage caused by stress, and ensuring normal metabolic function (Gallie, 2013). A study conducted on two wheat species showed that endogenous ascorbic acid content increased because of cold stress (Kader et al., 2011). An explanation for the elevated ascorbic acid levels could be an expression of plant stress resistance (Sivaci et al., 2014). While inositol is considered an essential vitamin-like ascorbic acid pathway substance that is converted to the TCA cycle through galactose metabolism, the plant body can synthesize inositol autonomously; if inositol metabolism is insufficient or impaired in plants, plant stress tolerance, growth, and development will be limited (Sang et al., 2017). In contrast, in the current study, vitamin and cofactor metabolism (inositol) after ramie crop succession played critical roles as intermediates of sugar, fatty acid, and amino acid metabolism. As shown in **Figures 5**, **S3**, one inositol metabolite was significantly upregulated in severe tillage soil damage stress, suggesting that ramie plants may reduce the concentration of reactive oxygen species through the ascorbic acid metabolic pathway, thereby mitigating free radical damage to plants. The antioxidant process involved in ascorbic acid is also an important defensive response of ramie in response to inheritance disorders. In contrast, in the present study, the metabolism of vitamins and cofactors (inositol) after ramie crop succession played a critical role as intermediates of sugar, fatty acid, and amino acid metabolisms. As shown in **Figure 5**; **Supplementary Tables S3**, one inositol metabolite was significantly upregulated in severe tillage soil damage stress, suggesting that ramie plants may reduce the concentration of reactive oxygen species through the ascorbic acid metabolic pathway, thereby mitigating free radical damage to plants. The antioxidant process involved in ascorbic acid is also an important defensive response of ramie in response to inheritance disorders. According to the correlation analysis with physiological indicators of ramie, inositol metabolites were negatively correlated with SD, BW, FOR, FY, and S-NP, with correlation coefficients of -0.811 -0.904 -0.917 -0.895 -0.836, respectively (**Table S4**). This indicated that traits such as SD, BW, FY, and S-NP of ramie reflected adverse effects when more inositol metabolites were secreted into the soil and that inositol metabolites can be used as a marker of disease.

As mentioned above, this study simultaneously elucidated that sinapic acid, jasmonic acid, glutamine, and myo-inositolmay be the main metabolites affecting ramie continuous cropping obstacles, and provided important insights into the mechanisms of the obstacles to continuous ramie cropping.

## 5 Conclusion

In summary, in the present study, metabolomics based on LC-MS and GC-MS was used to systematically investigate the metabolite composition, abundance, and metabolic pathways in ramie following continuous ramie cropping, revealing significant changes in the levels of phenylpropane compounds, fatty acids, amino acids, and other important metabolites. Furthermore, we demonstrated the enhanced resistance of ramie plants to the ramie continuous cropping barrier, predicted that these metabolites may function in concert to establish a complex metabolic network for mitigating ramie continuous cropping barriers, and provided important insights into the mechanism of the obstacles to continuous ramie cropping.

## Data availability statement

The original contributions presented in the study are included in the article/**Supplementary Material**. Further inquiries can be directed to the corresponding authors.

## Author contributions

SZ designed the experiments. QL and YW and provided experimental methods. QG and GL performed the research, YF, TL and XW analyzed the data and wrote the manuscript and reviewed the manuscript. All authors contributed to the article and approved the submitted version.

## References

[B1] Bai Y. (2017). Effect of potential allelochemicals from ramieon rhizosphere microbial diversity andphysiology and biochemistry of ramie [D]. Hunan Agricultural University.

[B2] Chen J. Y. Wen P. F. Kong W. F. Pan Q. H. Wan S. B. Huang W. D. (2006). Changes and subcellular localizations of the enzymes involved in phenylpropanoid metabolism during grape berry development. J. Plant Physiol. 163 (2), 115–127. doi: 10.1016/j.jplph.2005.07.006 16399002

[B3] Cui J. Q. Sun H. B. Sun M. B. Liang R. T. Jie W. G. Cai B. Y. (2018). Effects of funneliformis mosseae on root metabolites and rhizosphere soil properties to continuously-cropped soybean in the potted-experiments. Int. J. Mol. Sci. 24, 19(8):2160. doi: 10.3390/ijms19082160 PMC6121952 30042347

[B4] Dhawi F. Datta R. Ramakrishna W. (2016). Mycorrhiza and heavy metal resistant bacteria enhance growth, nutrient uptake and alter metabolic profile of sorghum grown in marginal soil. Chemosphere 157, 33–41. doi: 10.1016/j.chemosphere.2016.04.112 27208643

[B5] Dong L. Xu J. Zhang L. Yang J. Liao B. Li X.. (2017). High-throughput sequencing technology reveals that continuous cropping of American ginseng results in changes in the microbial community in arable soil. Chin. Med. 12, 18. doi: 10.1186/s13020-017-0139-8 28680459 PMC5496220

[B6] Fu H. D. Zhang G. X. Zhang F. Sun Z. P. Geng G. M. Li T. L. (2017). Effects of continuous tomato monoculture on soil microbial properties and enzyme activities in a solar greenhouse. Sustainability 9, 317. doi: 10.3390/su9020317

[B7] Gallie D. R. (2013). The role of l-ascorbic acid recycling in responding to environmental stress and in promoting plant growth. J. Exp. Bot. 64 (2), 433–443. doi: 10.1093/jxb/ers330 23162122

[B8] Han G. Z. (2017). Evolution of jasmonate biosynthesis and signaling mechanisms. J. Exp. Bot. 68 (6), 1323–1331. doi: 10.1093/jxb/erw470 28007954

[B9] Huang W. Sun D. Wang R. An Y. (2021). Integration of transcriptomics and metabolomics reveals the responses of sugar beet to continuous cropping obstacle. Front. Plant Sci. 12. doi: 10.3389/fpls.2021.711333 PMC8578061 34777408

[B10] Kader D. Z. A. Saleh A. A. H. Elmeleigy S. A. Dosoky N. S. (2011). Chilling-induced oxidative stress and polyamines regulatory role in two wheat varieties. J. Taibah Univ. Sci. 5, 14–24. doi: 10.1016/S1658-3655(12)60034-X

[B11] Kaur J. Singh J. P. (2014). Long-term effects of continuous cropping and different nutrient management practices on the distribution of organic nitrogen in soil under rice-wheat system. Plant Soil Environ. doi 60, 63–68. doi: 10.17221/440/2013-Pse

[B12] Li W. Liu Q. Chen P. (2018). Effect of long-term continuous cropping of strawberry on soil bacterial community structure and diversity. J. Integr. Agric. 17 (11), 2570–2582. doi: 10.1016/S2095-3119(18)61944-6

[B13] Li J. Li Y. Tian Y. Qu M. Zhang W. Gao L. (2017). Melatonin Has the Potential to Alleviate Cinnamic Acid Stress in Cucumber Seedlings. Frontiers in plant science, 8, 1193. doi: 10.3389/fpls.2017.01193 28751899 PMC5508022

[B14] Li J. Shu X. Xu J. Su S. M. Chan U. I. Mo L.. (2022). S100A9-CXCL12 activation in BRCA1-mutant breast cancer promotes an immunosuppressive microenvironment associated with resistance to immunotherapy. Nat. Commun. 13 (1), 1481. doi: 10.1038/s41467-022-29151-5 35304461 PMC8933470

[B15] Liu X. Zhang J. L. Gu T. Y. Zhang W. M. Shen Q. R. Yin S. X.. (2014). Microbial community diversities and taxa abundances in soils along a seven-year gradient of potato monoculture using high throughput pyrosequencing approach. PloS One 9, 86610. doi: 10.1371/journal.pone.0086610 PMC3907449 24497959

[B16] Lyu J. Jin N. Meng X. Jin L. Wang S. Xiao X.. (2022). Exogenous silicon alleviates the adverse effects of cinnamic acid-induced autotoxicity stress on cucumber seedling growth. Front. Plant Sci. 13. doi: 10.3389/fpls.2022.968514 PMC9399776 36035700

[B17] Lyu X. Ng K. R. Mark R. Lee J. L. Chen W. (2018). Comparative metabolic profiling of engineered saccharomyces cerevisiae with enhanced flavonoids production. J. Funct. Foods 44, 274–282. doi: 10.1016/j.jff.2018.03.012

[B18] Miska J. Rashidi A. Lee-Chang C. Gao P. Lopez-Rosas A. Zhang P.. (2021). Polyamines drive myeloid cell survival by buffering intracellular pH to promote immunosuppression in glioblastoma. Sci. Adv. 7 (8):eabc8929. doi: 10.1126/sciadv.abc8929 33597238 PMC7888943

[B19] Nićiforović N. Abramovič H. (2014). Sinapic acid and its derivatives: natural sources and bioactivity. Compr. Rev. Food Sci. Food Saf. 13 (1), 34–51. doi: 10.1111/1541-4337.12041 33412688

[B20] Oliva M. Guy A. Galili G. Dor E. Schweitzer R. Amir R.. (2021). Enhanced production of aromatic amino acids in tobacco plants leads to increased phenylpropanoid metabolites and tolerance to stresses. Front. Plant Sci. 11. doi: 10.3389/fpls.2020.604349 PMC7835393 33510749

[B21] Paupière M. J. van Heusden A. W. Bovy A. G. (2014). Themetabolic basis of pollen thermo-tolerance:perspectives for breeding. Metabolites 4, 889–920. doi: 10.3390/metabo4040889 25271355 PMC4279151

[B22] Perez-Brandan C. Huidobro J. Grumberg B. Scandiani M. M. Luque A. G. Meriles J. M.. (2014). Soybean fungal soil-borne diseases: a parameter for measuring the effect of agricultural intensification on soil health. Can. J. Microbiol. 60 (2), 73–84. doi: 10.1139/cjm-2013-0792 24498984

[B23] Raval S. S. Mahatma M. K. Chakraborty K. Bishi S. K. Singh A. L. Rathod K. J.. (2017). Metabolomics of groundnut (Arachis hypogaea l.) genotypes under varying temperature regimes. Plant Growth Regul. 84 (3), 493–505. doi: 10.1007/s10725-017-0356-2

[B24] Rehman M. Fahad S. Saleem M. Hafeez M. Rahman M. Liu F.. (2020). Red light optimized physiological traits and enhanced the growth of ramie (Boehmeria nivea l.). Photosynthetica 58 (4), 922–931. doi: 10.32615/ps.2020.040

[B25] Rizhsky L. Liang H. Shuman J. Shulaev V. Davletova S. Mittler R. (2004). When defense pathways Collide.The response of arabidopsis to a combination of drought and heat stress. Plant Physiol. 134, 1683–1696. doi: 10.1104/pp.103.033431 15047901 PMC419842

[B26] Sanchez D. H. Lippold F. Redestig H. Hannah M. A. Erban A. Kra¨mer U.. (2008). Integrative functional genomics of salt acclimatization in the model legume lotus japonicas. Plant J. 53 (6), 973–987. doi: 10.1111/j.1365-313X.2007.03381.x 18047558

[B27] Sang S. Chen Y. Yang Q. Wang P. (2017). Arabidopsis inositol polyphosphate multikinase delays flowering time through mediating transcriptional activation of FLOWERING LOCUS c. J. Exp. Bot. 68 (21-22), 5787–5800. doi: 10.1093/jxb/erx397 29161428 PMC5854132

[B28] Sivaci A. Kaya A. Duman S. (2014). Effects of ascorbic acid on some physiological changes of pepino (Solanum muricatum ait.) under chilling stress. Acta Biol Hungarica 65 (3), 305–318. doi: 10.1556/ABiol.65.2014.3.7 25194734

[B29] Son S. Y. Kim N. K. Lee S. M. (2016). Metabolite fingerprinting, pathway analyses, and bioactivity correlations for plant species belonging to the cornaceae, fabaceae, and *Rosaceae* families. Plant Cell Rep 35 (9), 1917–1931. doi: 10.1007/s00299-016-2006-y 27344340

[B30] Song X. Huang L. Li Y. Zhao C. Tao B. Zhang W. (2022). Characteristics of soil fungal communities in soybean rotations. Front. Plant Sci. 13. doi: 10.3389/fpls.2022.926731 PMC9260669 35812925

[B31] Subhashini D. V. Kumar H. (2019). Effect of long-term application of mineral fertilizers and FYM on microbial dynamics, yield and quality of FCV tobacco (*Nicotiana tabacum*) grown in vertisols. Indian J. Agric. Sci. 89, 1328–1333. doi: 10.56093/ijas.v89i8.92867

[B32] Van Wyk D. A. B. Adeleke R. Rhode O. H. J. Bezuidenhout C. C. Mienie C. (2017). Ecological guild and enzyme activities of rhizosphere soil microbial communities associated with bt-maize cultivation under field conditions in north West province of south Africa. J. Basic Microbiol. 57, 781–792. doi: 10.1002/jobm.201700043 28731210

[B33] Wang S. Cheng J. Li T. Liao Y. (2020). Response of soil fungal communities to continuous cropping of flue-cured tobacco. Sci. Rep. 10, 19911. doi: 10.1038/s41598-020-77044-77048 33199813 PMC7669846

[B34] Wang R. Liu J. Jiang W. Ji P. Li Y. (2022). Metabolomics and microbiomics reveal impacts of rhizosphere metabolites on alfalfa continuous cropping. Front. Microbiol. 13. doi: 10.3389/fmicb.2022.833968 PMC9069006 35531271

[B35] Wang F. Xiao J. Zhang Y. Li R. Liu L. Deng J. (2021). Biocontrol ability and action mechanism of bacillus halotolerans against botrytis cinerea causing grey mould in postharvest strawberry fruit. Postharvest Biol. Technol. 174, 111456. doi: 10.1016/j.postharvbio.2020.111456

[B36] Wang L. G. Ye C. L. Chen J. Li J. J. Luo J. J. (2022). Effects of continuous cropping on bacteria community in oil flax soil. Agr. Res. Arid Areas 40, 70–75. doi: 10.7606/j.issn.1000-7601.2022.01.08

[B37] Wang Y. Zhu S. Liu T. Guo B. Li F. Bai X. (2020). Identification of the rhizospheric microbe and metabolites that led by the continuous cropping of ramie (Boehmeria nivea l. gaud). Sci. Rep. 10 (1), 20408. doi: 10.1038/s41598-020-77475-3 33230149 PMC7683709

[B38] Xu M. Galhano R. Wiemann P. Bueno E. Tiernan M. Wu W.. (2012). Genetic evidence for natural product-mediated plant-plant allelopathy in rice (Oryza sativa). New Phytol. 193 (3), 570–575. doi: 10.1111/j.1469-8137.2011.04005.x 22150231 PMC3257406

[B39] Xu J. Zhang Z. Li X. Wei J. Wu B. (2019). Effect of nitrous oxide against botrytis cinerea and phenylpropanoid pathway metabolism in table grapes. Sci. Hortic. 254, 99–105. doi: 10.1016/j.scienta.2019.04.061

[B40] Yang C. Hao R. J. Du X. D. Wang Q. H. Deng Y. W. Sun R. J.. (2018). GC–TOF/MS-based metabolomics studies on the effect of protein sources in formulated diet for pearl oyster pinctada fucata martensii. Aquaculture 486, 139–147. doi: 10.1016/j.aquaculture.2017.12.020

[B41] Zhang T. Gao C. Yue Y. Liu Z. Ma C. Zhou G.. (2017). Time-course transcriptome analysis of compatible and incompatible pollen-stigma interactions in brassica napus l. Front. Plant Sci. 8. doi: 10.3389/fpls.2017.00682 PMC5413569 28515735

[B42] Zhao X. Dong Q. Han Y. Zhang K. Shi X. Yang X.. (2022). Maize/peanut intercropping improves nutrient uptake of side-row maize and system microbial community diversity. BMC Microbiol. 22, 14. doi: 10.1186/s12866-021-02425-6 34996375 PMC8740425

[B43] Zhao L. Huang Y. Keller A. A. (2018). Comparative metabolic response between cucumber (Cucumis sativus) and corn (Zea mays) to a Cu(OH)_2_ nanopesticide. J. Agric. Food Chem. 66 (26), 6628–6636. doi: 10.1021/acs.jafc.7b01306 28493687

[B44] Zhu S. Wang Y. Xu X. Liu T. Wu D. Zheng X.. (2018). Potential use of high-throughput sequencing of soil microbial communities for estimating the adverse effects of continuous cropping on ramie (Boehmeria nivea l. gaud). PloS One 13 (5), e0197095. doi: 10.1371/journal.pone.0197095 29750808 PMC5947917

