# Supplementary material for: Untargeted metabolomics reveal rhizosphere metabolites mechanisms on continuous ramie cropping
Source: Front Plant Sci. 2023 Aug 22;14:1217956. doi: 10.3389/fpls.2023.1217956 (PMC10477603; doi:10.3389/fpls.2023.1217956)
Supplement: Supplementary file 1 [file DataSheet_1.docx]

*Supporting materials for*

**Metabolomics reveals molecular mechanisms of continuous cropping obstacle in ramie (*Boehmeria nivea* L. Gaud)**

Yafen Fu^1†^, Tongying Liu^1^, Xin Wang^1^, Guang Li^1^, Siyuan Zhu^1*^

^†^ These authors have contributed equally to this work and share the first authorship.

^1^ Institute of Bast Fiber Crops, Chinese Academy of Agricultural Sciences, Changsha, China.

*Correspondence: Si Yuan Zhu [zhusiyuan@caas.cn](mailto:zhusiyuan@caas.cn)


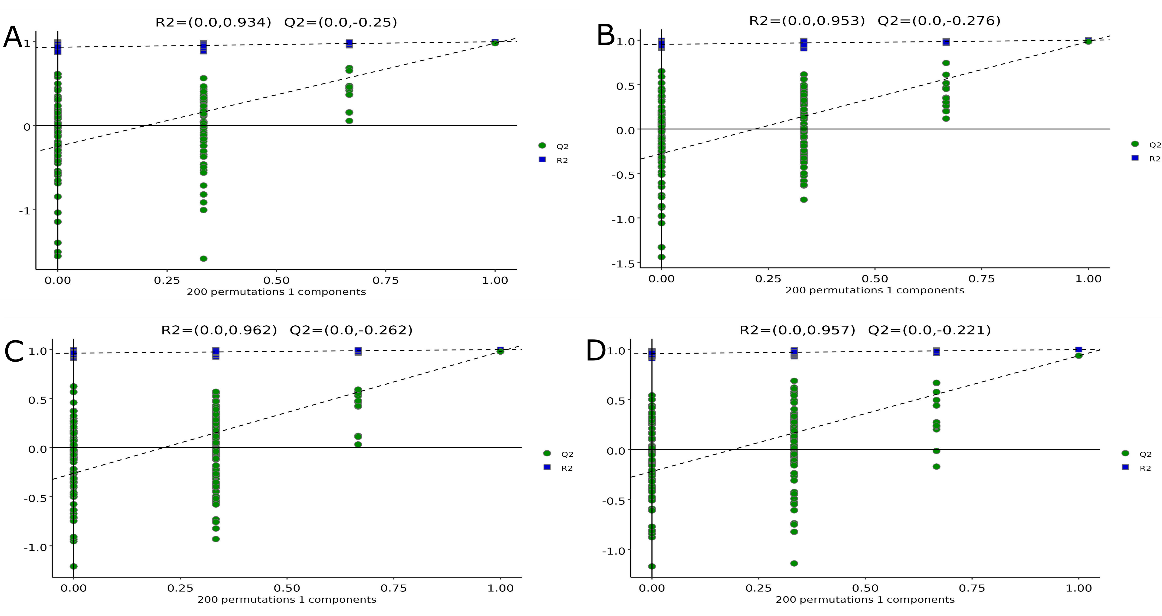


**Figure S1**. The corresponding permutation test plots of LC-MS of four continuous ramie soils. (A) XZQG; (B) JZ; (C) DJY; (D) GXD.


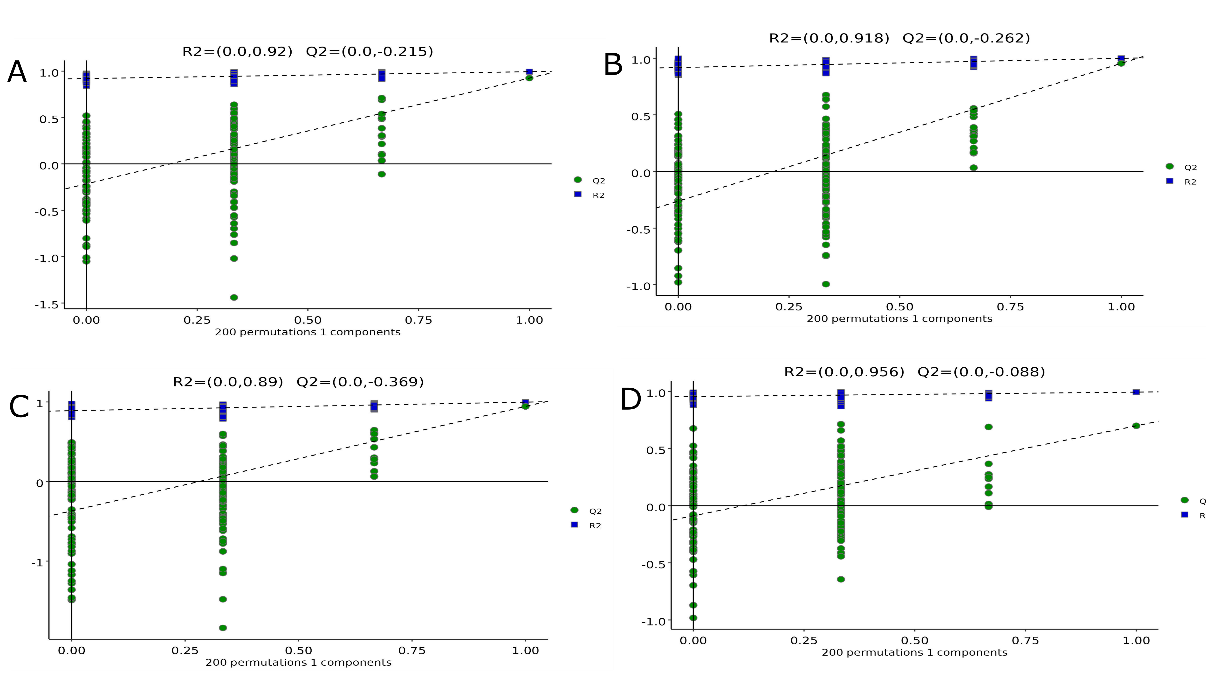


**Figure S2**. The corresponding permutation test plots of GC-MS of four continuous ramie soils. (A) XZQG; (B) JZ; (C) DJY; (D) GXD.

**
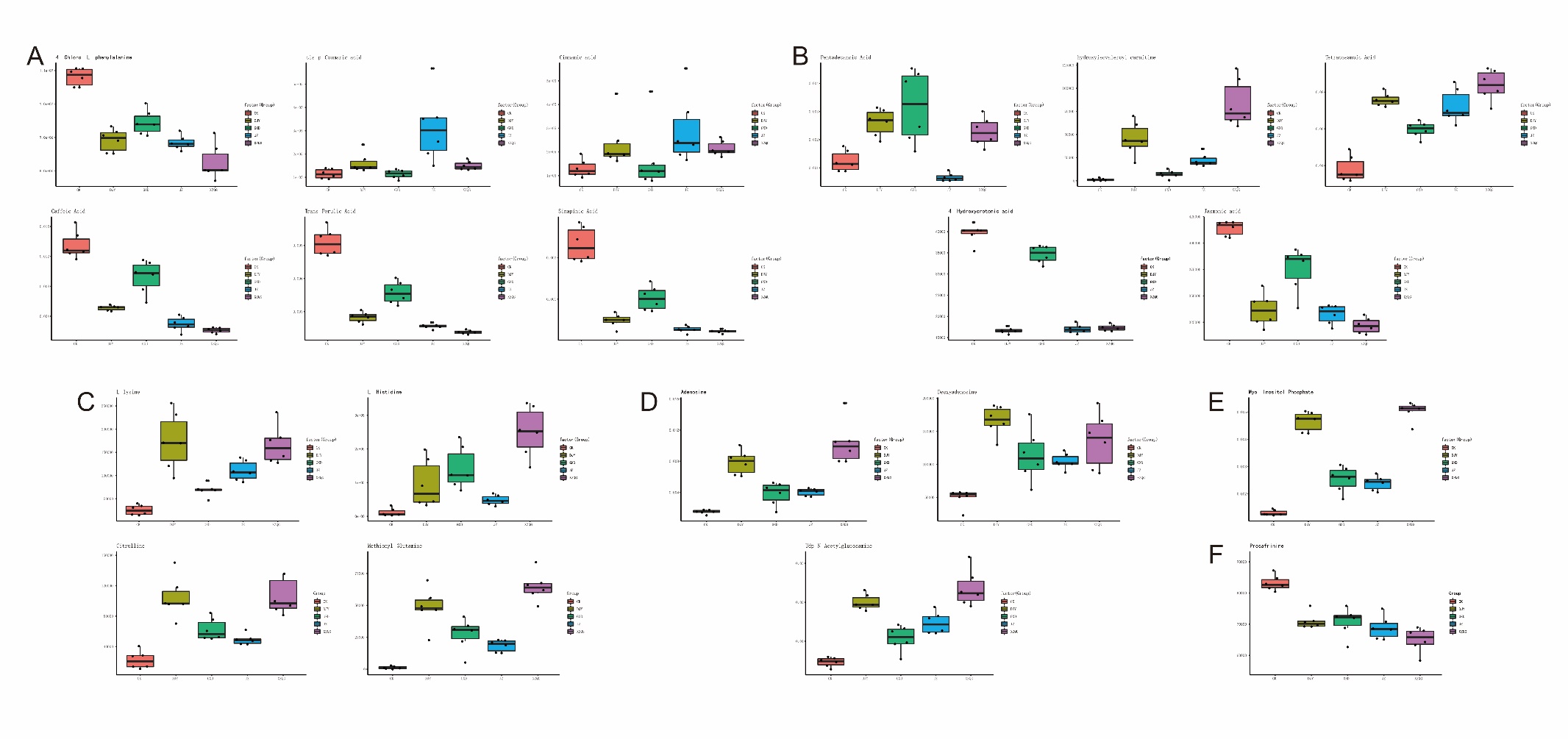
Figure S3**. Box-whisker plots showing the relative abundance of metabolites significantly varying in ramie continuous cropping soil, and Asterisks indicates significant differences (P≤0.05) according to Studentś t-test. (A) Phenylpropane metabolic pathway; (B) Fatty acid metabolism; (C) Amino acids and derivatives metabolism; (D) Amino sugar and nucleotide metabolism; (E) Ascorbate aldarate metabolism; ( F) Lipids metabolism.
